# Acute Kidney Injury Due to Leukemic Infiltration in a Patient with Chronic Lymphocytic Leukemia

**DOI:** 10.4274/tjh.galenos.2020.2020.0595

**Published:** 2021-06-01

**Authors:** Gizem Kumru Şahin, Hasan Emre Kocabay, Saba Kiremitçi, Osman İlhan, Kenan Keven

**Affiliations:** 1 Ankara University Faculty of Medicine, Department of Nephrology, Ankara, Turkey; 2 Ankara University Faculty of Medicine, Department of Internal Medicine, Ankara, Turkey; 3 Ankara University Faculty of Medicine, Department of Medical Pathology, Ankara, Turkey; 4 Ankara University Faculty of Medicine, Department of Hematology, Ankara, Turkey

**Keywords:** Acute kidney injury, Chronic lymphocytic leukemia, Leukemic infiltration

## To the Editor,

The kidneys are among the organs most affected by leukemic involvement in chronic lymphocytic leukemia (CLL) patients in postmortem autopsy series (63%-90%) [1,2]. However, leukemic infiltration (LI) of the kidneys has been a rare cause of renal impairment in CLL patients [3,4]. Here we describe a case of CLL presenting with severe acute kidney injury (AKI) due to LI.

**Figure 1:**
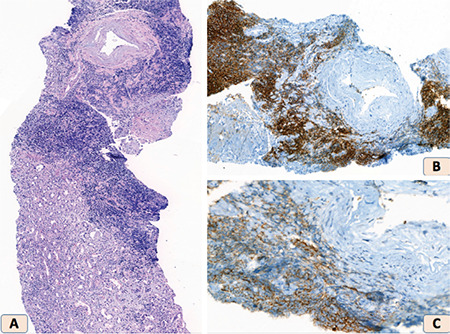
**(A)** Diffuse monomorphic neoplastic lymphocytic infiltrate in the renal tissue (H&E, 200^x^). **(B)** Lymphocytic infiltration showing immunopositivity for CD20 (immunohistochemistry, 300^x^). **(C)** Lymphocytic infiltration showing immunopositivity for CD5 (immunohistochemistry, 300^x^).

A 72-year-old white male without any renal disease presented with general malaise and weight loss. He had been diagnosed with stage B(1) B-CLL four years before and was treated with a bendamustine and rituximab protocol. Physical examination revealed general lymphadenopathy and hepatomegaly. Complete blood count findings were as follows: hemoglobin, 11.3 g/dL; leukocytes, 411.4x10^9^/L (lymphocytes: 60%); platelets, 247x10^9^/L. The blood ﬁlm appearances were suggestive of CLL. The following biochemical tests were abnormal: blood urea nitrogen, 44 mg/dL (normal: 8-22); serum creatinine (sCr), 6.16 mg/dL (normal: 0.7-1.3); CKD-EPI estimated-glomerular filtration rate, 8 mL/min/1.73 m^2^ (normal: >60). His urine had no leukocytes or casts, with 3 erythrocytes/field and proteinuria in the non-nephrotic range (1779 mg/24 h). Serological tests for hepatitis B, hepatitis C, HIV, and autoimmune kidney disorders were negative. Serum-free light chain (sFLC) kappa was 317.5 mg/dL (normal: 3.3-19.4) and sFLC lambda was 22.2 mg/dL (normal: 5.71-26.3), but no monoclonal bands in serum or urine immunofixation were detected. Renal ultrasound showed normal-sized kidneys with no evidence of obstructive nephropathy. A renal biopsy was performed, which demonstrated heavy infiltration of diffuse monomorphic neoplastic lymphocytes in the interstitium (Figure 1A). Six of 21 glomeruli were globally sclerotic and acute tubular necrosis and disruption were seen. Immunohistochemistry revealed that these infiltrative cells were positive for CD20 and CD5 (Figures 1B and 1C) without any amyloid or light chain deposition. The clinical picture was consistent with AKI due to LI of the kidneys. Therapeutic leukapheresis and treatment with high-dose methylprednisolone and rituximab were administered. His renal functions were restored with no need to perform hemodialysis and complete response in CLL was achieved (leukocytes: 10.3x10^9^/L). After a year of follow-up, his renal function had improved to sCr of 2.04 mg/dL, and a complete blood count revealed hemoglobin of 14.7 g/dL, leukocytes of 9.15x10^9^/L, and platelets of 294x10^9^/L.

Besides LI, renal impairment in CLL patients can be associated with prerenal azotemia, thrombotic microangiopathy, acute tubular necrosis, acute interstitial nephritis, uric acid nephropathy, light chain nephropathy, amyloidosis, obstructive nephropathy, glomerulonephritis, and cryoglobulinemia [5]. LI of the kidneys is common in CLL, but it is unlikely to be associated with severe AKI [4]. Although the mechanism of this clinical presentation is unclear, it was suggested that the compression of the tubular lumen and microvasculature by CLL cells may cause intrarenal obstruction and ischemia [6]. No clear association was demonstrated between CLL stage, intensity of interstitial infiltration, and severity of renal impairment [7]. Relapsed or refractory CLL and presentation with AKI are supportive for LI as the primary cause of renal impairment [6,8]. After exclusion of other causes, kidney biopsy should be performed to confirm the diagnosis. Significant improvement in renal function was reported after CLL treatment in a majority of patients [8,9]. Our case shows that LI of the kidneys can cause AKI as the initial manifestation of CLL and physicians should be aware of the atypical presentations of the disease.
